# Antifungal metabolites, their novel sources, and targets to combat drug resistance

**DOI:** 10.3389/fmicb.2022.1061603

**Published:** 2022-12-02

**Authors:** Megha Choudhary, Vijay Kumar, Bindu Naik, Ankit Verma, Per Erik Joakim Saris, Vivek Kumar, Sanjay Gupta

**Affiliations:** ^1^Himalayan School of Biosciences, Swami Rama Himalayan University, Dehradun, India; ^2^Department of Life Sciences (Food Technology & Nutrition), Graphic Era (Deemed to be University), Dehradun, India; ^3^Department of Microbiology, Faculty of Agriculture and Forestry, University of Helsinki, Helsinki, Finland

**Keywords:** antifungal agents, novel targets, drug resistant, unusual habitats, peptides

## Abstract

Excessive antibiotic prescriptions as well as their misuse in agriculture are the main causes of antimicrobial resistance which poses a growing threat to public health. It necessitates the search for novel chemicals to combat drug resistance. Since ancient times, naturally occurring medicines have been employed and the enormous variety of bioactive chemicals found in nature has long served as an inspiration for researchers looking for possible therapeutics. Secondary metabolites from microorganisms, particularly those from actinomycetes, have made it incredibly easy to find new molecules. Different actinomycetes species account for more than 70% of naturally generated antibiotics currently used in medicine, and they also produce a variety of secondary metabolites, including pigments, enzymes, and anti-inflammatory compounds. They continue to be a crucial source of fresh chemical diversity and a crucial component of drug discovery. This review summarizes some uncommon sources of antifungal metabolites and highlights the importance of further research on these unusual habitats as a source of novel antimicrobial molecules.

## Introduction

**Figure 1:**
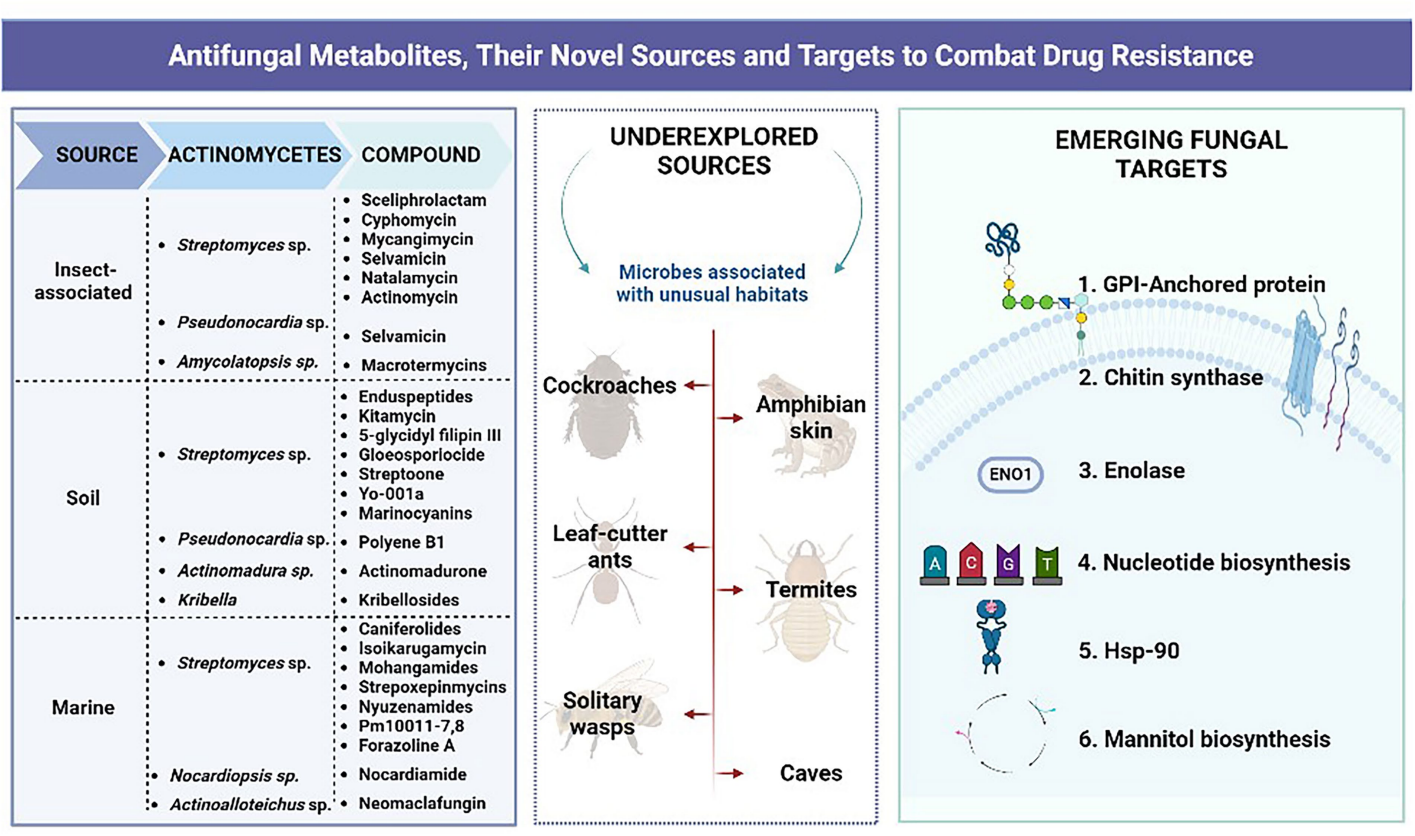
Graphical abstract.

Fungal diseases are already a global threat that is getting worse in the time of the ongoing COVID-19 pandemic, especially in developing countries. This pandemic is a troubling reminder of the fact that vulnerability still exists in the modern world. Even today, communicable diseases continue to be the world’s leading cause of death (WHO, 2019). Unfortunately, healthcare authorities have underestimated some of these microbial hazards in the past, even though they endanger the lives of millions of people every year. An important example of such overlooked diseases is fungal infections. Even with significant developments in the field of medicine in the past few decades, the spread of fungal infections is continuously on the rise. Furthermore, the long-term therapeutic use of antifungal medicines in patients has led to the emergence of multi-drug resistant fungal strains, including the particularly aggressive, *Candida auris*. The field of clinical mycology is dynamic and continually shifting. New risk factors for atypical mycoses have emerged as a result of a new therapy for malignant and autoimmune disorders. In transplant patients or people with cancer, the use of antifungal agents (such as triazoles or echinocandin) has been successful in reducing the occurrence of invasive fungal diseases but at the same time, has increased the risk of infections caused by non-fumigatus *Aspergillus* sp. in such patients (Husain and Camargo, 2019). There has been an emergence of azole-resistant in clinical and environmental isolates because of the widespread use of triazole insecticides in agriculture (Price et al., 2015). *Candida auris*, a new yeast species that was unknown barely a decade ago, is now spreading across the globe causing massive healthcare-related outbreaks in about 40 countries on 6 continents (Du et al., 2020). There has also been a recurrence of a once-rare fungal infection caused by the opportunistic *Fusarium* sp. in immunocompromised patients worldwide due to developing resistance to various antifungal drugs (Taj-Aldeen, 2017). One important reason contributing to the dramatic rise of fungal infections is the increase in the vulnerable population, such as the elderly, critically ill, or immunocompromised individuals. The rising figures of AIDS, cancer, and transplant patients along with the widespread use of immune-modulating medications and antibiotics, are some risk factors for the spread of FIs (Friedman and Schwartz, 2019; Lockhart and Guarner, 2019). Also, as medical equipment like catheters become more widely used, the danger of biofilm formation increases (Khatoon et al., 2018). Biofilms are a collection of highly diverse and well-organized cells that protect against physical and chemical threats. Biofilms are generally resistant to present treatments and are thought to be a major contributor to the high mortality rates in invasive fungal infections (Pierce et al., 2015). In addition to their direct effects on people, fungi also endanger food security by wreaking havoc on the crops that provide food for billions of people and by creating chemicals that contaminate food sources and cause cancer (Fisher et al., 2012). Mass deaths of bats and amphibians are endangering biodiversity, and an unusual number of fungal diseases have recently been responsible for the extinction of wild species (Fisher et al., 2020). As global temperatures rise and pests and pathogens migrate northward, with fungi leading the way, climate change is likely to make matters worse (Lazar et al., 2022). The rate of developing antifungals has been much slower as compared to that of antibacterial agents. One of the main reasons for this is the fact that fungi are eukaryotic organisms and most agents that have antifungal properties are also toxic to their eukaryotic host, i.e., humans. The key requirement for an antifungal drug is that the eukaryotic machinery of the fungal pathogen is selectively disabled or destroyed while causing little or no damage to the host cellular activities (Odds et al., 2003). Despite this limitation, several agents have been developed in the past along with numerous ongoing studies. According to the figures from 2012, just 0.005 percent of new pharmaceuticals made from synthetic molecules are successful, compared to 0.6 percent and 1.6 percent, respectively, for the development of complete natural items and natural microbial products (Bérdy, 2012). The majority of medications on the market today were created through the synthesis or semi-synthesis of natural compounds and microbes continue to be a valuable source for the creation of new natural products (Newman and Cragg, 2020). Gene modification can be used to alter the bioactive molecules collected from bacteria to create better or ideal pharmaceuticals, which are then mass manufactured. The overview of the review is given in Figure 1.

### Status of antifungals and drug resistance in fungi

Many superficial mycoses have been treated with a range of topical medicines that have had moderate efficacy in reducing common infections over the last century. The fact that there are limited antifungal medications available to treat systemic fungal infections increases the impact of fungus on human health (Perfect, 2017). There are only four primary classes of available antifungal drugs, which include- polyenes, flucytosine, azoles, and echinocandins (Carmona and Limper, 2017). Many opportunistic fungi such as *Candida auris* and *Lomentospora prolificans* are innately resistant to many antimycotics (Arastehfar et al., 2020). Some species, like *C. glabrata*, have strong intrinsic resistance to antifungals like azoles, while other species such as *C. albicans*, can acquire resistance to antifungals after being exposed to them, mainly during preventive treatments (Arastehfar et al., 2020). Despite the limited number of antimicrobials available, microorganisms have a remarkable capacity to develop resistance to them, posing a serious threat to public health. Fungal pathogens, which can only be treated with a limited number of antifungal medications and produce catastrophic invasive infections, are of particular concern. The rates of resistance for fungal infections vary by geography, species, and accessible treatments; nonetheless, the spread of drug-resistant fungus species is global. In general, fungal pathogens’ innate and acquired drug resistance strategies involve lowering the effective drug concentration, changing the drug target, or rerouting antifungal toxicity through metabolic change (Sanglard, 2016).

Some of the challenges that hinder antifungal drug development can be summarized as follows:

- Current antifungal medicines have not been as efficient to reduce mortality caused by fungal infections and the efficacy of available antifungal therapies has reached a stage of plateau.
- Also, there has to be a larger focus on developing drugs having faster fungicidal efficacy to improve clinical outcomes. The current treatment course for typical antifungal medicines is far too long, posing the risk of inadequate short-term fungicidal effectiveness, decreased patient compliance and/or tolerance, or even the formation of direct antifungal drug resistance (Cuenca-Estrella, 2014).
- There is a need to broaden the antifungal spectrum to include some developing, extremely drug-resistant fungi (like *Candida auris*) that are increasingly being seen in hospitalized patients who are immunosuppressed due to other serious underlying conditions.
- To boost potency and prevent the establishment of drug resistance, it is critical to have an optimum mix of therapeutic agents or classes. Some antifungal drug classes, such as azoles and echinocandins, are seeing an increase in resistant strains (Smith et al., 2015).

### Potential sources of novel antifungals

#### Actinomycetes

Many novel actinobacteria have been published with the hypothesis that novel strains from previously undiscovered sources could be great targets for the discovery of bioactive substances (van Bergeijk et al., 2020). Table 1 lists the novel natural antifungal compounds that have been isolated from actinomycetes since 2011. Several recent research on natural product discovery has concentrated on the examination of insect-microbe symbioses and especially, interactions between insects and actinobacteria because bacterial symbionts need small molecules to communicate with the host or take part in host defence (Chouvenc et al., 2018; Simon et al., 2022). The associations between endosymbiotic bacteria with arthropods can vary from facultative to obligate mutualisms; these bacteria can assist with nutrient uptake, promote resistance to plant secondary metabolite, and chemical pollutant and pesticide detoxification (Zhao et al., 2022), while some symbionts exude antifungals to inhibit the proliferation of the arthropods’ fungal antagonists (Kett et al., 2021). Firebugs and the European beewolf, which harbors antibiotic-producing *Streptomyces* in their antennae to help protect their larvae from fungus infections, are two examples of the importance of defensive secondary metabolites in insect-Actinobacteria symbiosis (Kaltenpoth et al., 2012; Salem et al., 2013). Similar to this, ants that cultivate fungi of the Attini species have symbiotic *Pseudonocardia* that aid in guarding the ants’ fungi against specific pathogens (Oh et al., 2009; Poulsen et al., 2010). Other insects, such as dung beetles (Scarabaeidae: *Copris tripartitus*), and fungus-growing termites, have also been documented to harbor insect-associated Actinobacteria (Kurtböke et al., 2015; Um et al., 2015).

**Table 1:** List of novel natural antifungal compounds isolated from actinomycetes (2011-present).

Name of compound	Class	Target	Organism	Source of organism	Activity against	Reference
Sceliphrolactam	Polyene	Cell membrane	*Streptomyces* sp.	*Sceliphron caementarium* wasp	*Candida albicans*	Oh et al., 2011
Neomaclafungin A	Macrolide	Glucan synthase	*Actinoalloteichus* sp. NPS702	Marine sediment	*Trichophyton mentagrophytes*	Sato et al., 2012
Natalamycin	Bicyclic macrolide	Hsp90	*Streptomyces* sp. M56	Termite-associated	*Pseudoxylaria* X802, *Termitomyces* T112	Kim et al., 2014
Forazoline A	Polyketide	Disruption of membrane	*Actinomadura sp.*	Marine invertebrate-associated	*Candida albicans*	Wyche et al., 2014
Isoikarugamycin, 28-N-methylikarugamycin	Polycyclic tetramic acid macrolactam	NA	*Streptomyces zhaozhouensis* CA185989	Marine sediment	*Aspergillus fumigatus, Candida albicans*	Lacret et al., 2014
5-Alkyl-1,2,3,4-tetrahydroquinolines	Quinoline	Cell membranes	*Streptomyces nigrescens HEK616* and	Soil, Human sputum	*Schizosaccharomyces pombe*	Sugiyama et al., 2015
			*Tsukamurella pulmonis TPB0596*			
Mohangamides A and B	Dilactone-tethered pseudodimeric peptide	Isocitrate lyase in glyoxylate cycle	*Streptomyces* sp. SNM55	Marine mudflat	*Candida albicans*	Bae et al., 2015
21-deoxy-bafilomycin A1	Polyene	Inhibiton of vacuolar-ATPase	*Streptomyces albolongus* YIM	Elephant dung	*Candida albicans*, *Candida parapsilosis*	Ding et al., 2016
			1,01,047		, *Cryptococcus neoformans*	
15-glycidyl filipin III	Polyene	Disruption of cell membrane	*Streptomyces lavenduligriseus*	Soil	*Candida albicans*	Yang et al., 2016
Enduspeptides A-F	Cyclic octa depsipeptide	NA	*Streptomyces* sp.	Coal mine soil	*Candida glabrata*	Chen et al., 2017
Actinomadurone	Polycyclic tetrahydroxanthone	Hsp inhibition	*Actinomadura* sp. BCC 35,430	Soil	*Curvularia lunata*, *Alternaria brassicicola*, *Colletotrichum capsici*, *Colletotrichum gloeosporioides*	Bunyapaiboonsri et al., 2017
Kitamycin C	Antimycin type macrolide	Inhibiton of cellular respiration	*Streptomyces antibioticus* strain 200–09	Soil	*Candida albicans*	Wang et al., 2017
Kribellosides A-D	Alkyl glyceryl ethers	RNA 5′-triphosphatase inhibitor	*Kribella* MI481-42F6	Soil	*Saccharomyces cerevisiae*	Igarashi et al., 2017
Nabscessins A and B	Aminocyclitol amide	NA	*Nocardia abscessus* IFM 10,029	Human intra-articular abscess	*Cryptococcus neoformans*	Hara et al., 2017
Marinocyanins A	Bromo-phenazinone	NA	*Streptomyces sp. HCCB11876*	Soil	*Candida albicans*	Asolkar et al., 2017
Macrotermycins A and D	Glycosylated polyketide	NA	*Amycolatopsis* sp. M39	Termite-associated	*Metarhizium anisopliae*, *Beauveria bassiana*	Beemelmanns et al., 2017
Polyene B1	Polyene	Ergosterol in cell membrane	*Pseudonocardia autotrophica*	Soil	*Candida albicans*	Kim et al., 2018
			KCTC9441			
Streptoone B	Linear polyketide	NA	*Streptomyces sp. SN0280*	Soil	*Phytophthora capsici*	Tian et al., 2017
Gloeosporiocide	Cyclic peptide	NA	*Streptomyces morookaense* AM25	Amazon soil	*Colletotrichum gloeosporioides*	Vicente dos Reis et al., 2019
Pm100117 and Pm100118	Glycosylated polyketides	NA	*Streptomyces caniferus* GUA-06-05-006A	Marine	*Candida albicans*	Pérez et al., 2016
Strepoxepinmycins A–D	Naphthoquinone	DNA damage or inhibition of protein synthesis	*Streptomyces sp. XMA39*	Marine	*Candida albicans*	Jiang YL. et al., 2018
Umezawamides A	Polycyclic macrolactam	NA	*Umezawaea sp. RD066910 +*	-	*Candida albicans*	Hoshino et al., 2018
			*Tsukamurella pulmonis TPB0596*			
10-deoxyfungichromin (WH02)	Polyene	NA	*Saccharothrix yanglingensis*	Cucumber root endophyte	*Valsa mali*	Wang H. et al., 2019
			Hhs.015			
Actinomycin D	Bicyclic chromopeptide lactone	Transcription inhibitor	*Streptomyces* sp. T33	Termite-associated	*Xylaria*	Yin et al., 2019
Caniferolides A–D	Polyol macrolide	NA	*Streptomyces caniferus* CA-271066	Marine	*Candida albicans*, *Aspergillus fumigatus*	Pérez-Victoria et al., 2019
Cyphomycin	Macrocyclic macrolide	NA	*Streptomyces* sp.	*Cyphomyrmex* sp. Ant	*Candida albicans*	Chevrette et al., 2019
Rubromycins CA1 and CA2	Aromatic polyketide	NA	*Streptomyces hyaluromycini* MB-PO13	*Molgula manhattensis* Sea Grape	*Candida albicans*	Harunari et al., 2019
Yo-001a	Oligomycin macrolide	NA	*Streptomyces sp. YO15-A001*	Soil	*Pyricularia oryzae*	Yamamoto et al., 2019
Nyuzenamides A and B	Bicyclic peptide	NA	*Streptomyces*	Marine	*Glomerella cingulate*,	Karim et al., 2021
					*Trichophyton rubrum*	

## Microorganisms associated with animal hosts

**Figure 2:**
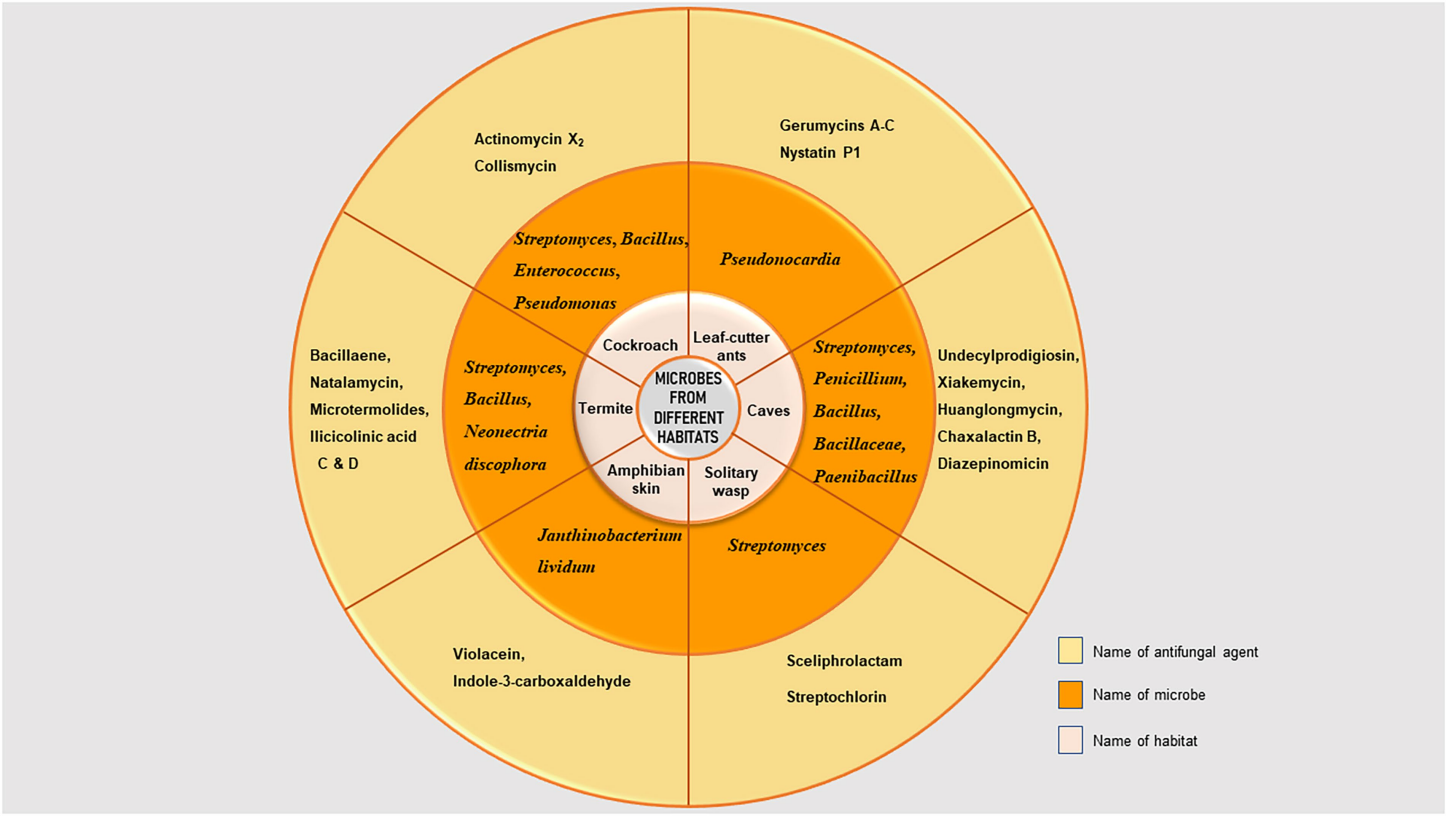
Distribution of microorganisms and their metabolites isolated from different habitats.

Because the host must endure exposure to these substances, bacteria found in associations with animal hosts are likely to generate antimicrobial chemicals that are less harmful than those found in soil bacteria. Figure 2 depicts the distribution of microorganisms and their metabolites from different habitats mentioned in this review.

### Cockroaches

Cockroaches have been found to carry members of several related bacterial families, most notably the Enterobacteriaceae, Staphylococcaceae, and Mycobacteriaceae species. The most often reported genera from the cockroach gut were *Streptomyces*, *Bacillus*, *Enterococcus*, and *Pseudomonas* (Guzman and Vilcinskas, 2020). Feces of the cockroach *Cryptocercus punctulatus* demonstrated antifungal action linked to compounds made by gut microorganisms (Rosengaus et al., 2013). A species of actinobacteria, *Streptomyces globisporus* WA5-2-37 that produces Actinomycin X_2_ and Collismycin, was just recently discovered in the intestine of *Periplaneta americana* (Chen et al., 2020). *Bacillus atrophaeus*, *Bacillus subtilis*, and *Pseudomonas reactans* are a few bacterial strains that have been successfully isolated from the gut of *Blatella germanica* and have the ability to limit the growth of entomopathogenic fungus *Beauveria bassiana* (Huang et al., 2013).

### Amphibian skin microbiome

Vertebrate skin microbiomes act as a defense against infections and can influence disease risk. Higher bacterial species richness, certain microbial community assemblages, and the presence of microorganisms that produce compounds that impede pathogen growth have all been linked to lower infection risk in vertebrates. Many amphibians’ population declines and extinctions have been attributed to the fungal pathogen *Batrachochytrium dendrobatidis* (Bd), which causes chytridiomycosis (Berger et al., 2016). Since these circumstances match with the pathogen’s ideal development range, chytridiomycosis has primarily afflicted tropical amphibian populations at high elevations and during cooler seasons (Sapsford et al., 2013). Amphibians have a variety of defence mechanisms, including the presence of skin bacteria that create antifungal metabolites. For example, the bacteria *Janthinobacterium lividum*, which lives in the salamander *Plethodon cinereus*, produces anti-Bd secondary metabolites such as violacein and indole-3-carboxaldehyde (Brucker et al., 2008).

### Termites

Communities of symbiotic microorganisms are found in insect digestive systems, and these communities are the focus of research on microbial diversity and new bioactive microbial compounds (Breznak, 2003). Fungus-farming termites belonging to the subfamily *Macrotermitinae*, cultivate basidiomycetes (genus *Termitomyces*) as their main source of food (Lever et al., 2015) and these basidiomycetes are constantly susceptible to parasitism by other fungi. The termites probably use a variety of tactics to manage such antagonists, with the production of antifungals being one among them (Um et al., 2013). The gut flora of the Macrotermitinae has so far been found to contain seven different bacterial phyla (Otani et al., 2014). *Bacillus* strains are the most prevalent and can produce antifungals among them. An initial analysis of an extract of the *Bacillus* strain cultures using liquid chromatography and mass spectrometry identified a significant secondary metabolite: Bacillaene, a polyene polyketide that is present in all strains and which inhibits the growth of *Pseudoxylaria*, *Trichoderma*, and *Fusarium* (Um et al., 2013). From the nests of the French Guiana termite *Nasutitermes corniger*, the fungus *Neonectria discophora* SNB-CN63 was isolated (Sorres et al., 2018) that produced Ilicicolinic acid C and D. These are non-macrocyclic, non-polycyclic polyketide that showed antifungal activities against *Trichophyton rubrum* and *Candida albicans* (Nirma et al., 2015). There is proof that the hindgut microbiota of the termite *Zootermopsis angusticollis* produces many functionally active b-1,3-glucanases with a probable involvement in fungal pathogen protection (Rosengaus et al., 2014). Termites also support the *Streptomyces* species, which makes the antifungal Natalamycin (Kim et al., 2014). Other antibiotics known as microtermolides A and B are produced by the *Streptomyces* strain connected to the fungus-farming termites (Carr et al., 2012).

### Solitary wasp

The solitary wasps *Sceliphron caementarium* and *Chalybion californicum* are associated with *Streptomyces* spp. that produce Streptochlorin, a range of Piericidin analogs, and other chemicals that protect their larvae against bacteria and fungi (Poulsen et al., 2011). Sceliphrolactam, an antifungal substance, was discovered from *Streptomyces* that was associated to the mud dauber wasp *Sceliphron caementarium* (Oh et al., 2011). The substance is a polyene macrocyclic lactam with antifungal action against *Candida albicans* that is resistant to amphotericin B (Oh et al., 2011). Kumar et al. (2012, 2014) also reported novel species of *Streptomyces* from solitary wasp producing antimicrobial agents showing promising activity against drug resistant bacterial and fungal pathogens.

### Leafcutter ants

The leaf cutter ants have many defense mechanisms against the risk of pathogenic infection of their garden fungus, including a tripartite mutualistic interaction in which they host bacteria that produce antibiotics on their bodies (Barke et al., 2010). Numerous of these bacteria have coevolved with their hosts, creating antifungals to inhibit parasitic fungi (*Escovopsis* spp. and allied taxa), and in exchange, the ants feed the bacteria through special exocrine glands inside elaborate cuticular crypts, which also provide the bacteria with their preferred microclimate (Currie et al., 2006). *L. gongylophorus*, a mutualist fungus, is the only source of nourishment for *Acromyrmex*, which also harbors *Pseudonocardia* bacteria in its metapleural glands (Heine et al., 2018). Another fungus, *Escovopsis*, parasitizes the *L. gongylophorus* cultivar (Yek et al., 2012). To suppress *Escovopsis*, the *Pseudonocardia* produce various variations of the broad-spectrum polyene antifungal nystatin P1 (Holmes et al., 2016). Additionally, it has recently been discovered that *Pseudonocardia* associated with the attines *Apterostigma dentigerum* and *Trachymyrmex cornetzi* create novel cyclic depsipeptide substances known as gerumycins A-C (Holmes et al., 2016).

### Microorganisms isolated from caves

Cave habitats encourage the development of antimicrobials due to their unique properties, such as darkness, high humidity, consistent low temperature, and lack of nutrition which influence the microflora that thrives there (Ghosh et al., 2017). Such extreme environments have caught the attention of researchers due to the presence of undiscovered microbial communities in these caves. Due to a scarcity of nutrients in cave habitats, microorganisms become more competitive and adopt survival tactics such as secreting metabolites (antibiotics and enzymes; Ghosh et al., 2017). Various caves have been studied for potential bioactive metabolites all over the world. The *Streptomyces* genus has been found in a variety of caves in various countries including Thailand (Nakaew et al., 2009a), Turkey (Yücel and Yamaç, 2010), Pakistan (Zada et al., 2016), India (Yogita et al., 2012), China (Jiang et al., 2015), Russia (Axenov-Gibanov et al., 2016; Voytsekhovskaya et al., 2018), Canada (Riquelme et al., 2017)^,^ and Poland (Herold et al., 2005). Other Actinobacteria genera have also been found in cave habitats including *Arthrobacter*, *Nocardia*, *Saccharothrix*, *Lentzea*, *Micrococcus*, *Nonomuraea*, *Spirillospora*, *Micromonospora*, *Microbacterium*, *Cryobacterium*, and *Lysinibacter* (Lee et al., 2000; Ningthoujam et al., 2009; Nakaew et al., 2009b). But only a few bioactive substances have been documented so far and the chemical characteristics of the majority of the reported metabolites released by particular bacteria are unknown. The identified bioactive substances include undecylprodigiosin (Stankovic et al., 2012), xiakemycin and huanglongmycin (Jiang et al., 2015; Jiang L. et al., 2018), chaxalactin B (Castro et al., 2018), diazepinomicin (Gosse et al., 2019) from *Streptomyces* sp., a mixture of antibiotics of *Streptomyces* sp., *Bacillus* sp. and *Bacillaceae* (Axenov-Gibanov et al., 2016), a mixture of polyene and non-polyene metabolites of *Streptomyces* sp. and *Penicillium* sp. (Belyagoubi et al., 2018), lanthipeptides, polymyxin B, paenicidin B, fusaricidin, tridecaptin, and colistin A of Paenibacillus (Baindara et al., 2020; Lebedeva et al., 2021), lipids of *Toxopsis calypsus* and *Phormidium melanochroun* (Lamprinou et al., 2015).

### Animal secretions

Exocrine glands in arthropods secrete a variety of secondary compounds. The outer cuticle of the insect serves as the first line of defence against fungal infection. The first spider AMPs, known as lycotoxins-I and II from Lycosa carolinensis, possessed both antibacterial and antifungal properties that were able to prevent the growth of bacteria and the *Candida glabrata* yeast (Yan and Adams, 1998). Venom can have neurotoxic effects, but it can also have analgesic, anticancer, antiarrhythmic, and most importantly, antibacterial properties. Spider venom contains salts, acylpolyamines, peptides, enzymes, amino acids, nucleotides, and low molecular mass molecules (Vassilevski et al., 2009). The venom of *Ornithoctonus hainana* also contained an antibacterial substance called Oh-defensin that was active against both Gram-positive and Gram-negative bacteria as well as fungi (Zhao et al., 2014). When extracted from the hemolymph of scorpions of the species *Androctonus australis*, the peptide androctonin was found to be effective against both bacteria and fungus (Ehret-Sabatier et al., 1996). Melectin and halictines are two antimicrobial substances that can be found in the venom of Hymenopterans (Slaninová et al., 2011). Ponericins from the venom of the ponerine ant *Pachicondyla gueldi* has been observed to be effective against bacteria and yeasts (Orivel et al., 2001). Unknown toxins in the venom of the paper wasp *Polistes flavus* are also effective against *Candida* and *Aspergillus niger* (Prajapati and Upadhyay, 2016). The antimicrobial properties of the venom of wasps and bees have also been demonstrated in some studies. For instance, the crude venom of the wasp *Vespa orientalis* effectively exhibited antifungal efficacy against the fungal strain *Candida albicans* (Farag and Swaby, 2018). The venom of *Pseudopolybia vespiceps* wasps contains another mastoparan peptide that exhibits substantial antifungal properties against *Candida albicans* and *Candida neoformans* (Silva et al., 2017). Other recently discovered peptides with certain antifungal activities obtained from natural sources, i.e., animal secretions or microbes, have been summarized in Table 2.

**Table 2:** Antifungal peptides from natural sources.

Origin	Organism	Name of peptide	Mode of action	Active against	Reference
Bacteria	*Lactobacillus paracasei*	Bacteriocin KC39	disturbing the cytoplasmic membrane through pore formation, or by cell wall degradation	*Aspergillus parasiticus, Penicillium expansum*	Shehata et al., 2018
	*Lactobacillus plantarum*	FPSHTGMSVPPP	NA	*Aspergillus* spp.*, P. roqueforti*	Muhialdin et al., 2016
	*Lactobacillus plantarum*	LR/14	NA	*Aspergillus niger, Penicillium chrysogenum*	Gupta and Srivastava, 2014
	*Bacillus amyloliquefaciens*	Fengycin	Binding to lipid layers and altering cell membrane structure and permeability	*Fusarium oxysporum f. sp. radicislycopersici*	Kang et al., 2020
	*Bacillus velezensis*	Iturin	Membrane lysis by pore formation, inhibiting the activity of cytochrome P450 and halogenase	*Fusarium oxysporum*, *Ralstonia solanacearum*	Cao et al., 2018
	*Bacillus thuringiensis* SF361	YvgO	NA	*Byssochlamys fulva*	Manns et al., 2015
	*Paenibacillus polymyxa* jsa-9	AMP-jsa9	Targets cell wall structure and metabolism	*Fusarium moniliforme*	Han et al., 2017
	*Enterococcus durans* A5-11	Durancins A5-11a, A5-11b	NA	*Fusarium culmorum*	Belguesmia et al., 2013
Fungi	*Fusarium graminearum*	FgAFP	NA	*Fusarium verticilloides, F. proliferatum*	Patiño et al., 2018
	*Monascus pilosus*	MAFP1	NA	*Fusarium* spp.	Tu et al., 2016
	*Neosartoria fischeri*	NFAP2	Plasma membrane disruption	*Saccharomyces cerevisiae*, *Aspergillus nidulans*	Tóth et al., 2016
	*Penicillium chrysogenum*	Pc-Arctin/PAFC	plasma membrane disintegration, induction of intracellular ROS	*Amanita longipes, Byssochlamys spectabilis*	Holzknecht et al., 2020
	*Penicillium citrinum*	PcPAF	NA	*Fusarium oxysporum*, *Alternaria longipes*	Wen et al., 2014
	*Penicillium digitatum*	PdAfpB	Cell wall integrity	*Fusarium oxysporum, P. expansum*	Garrigues et al., 2017
	*Penicillium expansum*	PeAfp A, B and C	Cell wall integrity	*Alternaria alternata, Aspergillus* spp.*, Byssochlamys* spp*., Fusarium* spp.,	Garrigues et al., 2018
				*Penicillium* spp.	
	*Penicillium chrysogenum*	PgAFP/PAFB	cell wall integrity pathway, the stress-related *rho1* gene	*Fusarium oxysporum, Aspergillus flavus*	Huber et al., 2018
	*Emericellopsis alkalina*	Emericellipsin A	Cell membrane	*Aspergillus niger, A. flavus*	Rogozhin et al., 2018
Animals (insects, mammals, amphibians)	*E. rubrofemoratus, Eumenes fraterculus* wasp	Eumenine mastoparan-ER (EMP-ER), (EMP-EF), Eumenitin-F	Membrane disruption	*Candida albicans*	Rangel et al., 2011
	*Centruroides sculpturatus* scorpion	BmKbpp2	NA	*Fusarium culmorum*	Zeng et al., 2012
	Human	Defensin HBD-3	Cell lysis	*Fusarium culmorum, Penicillium expansum, Aspergillus niger*	Thery et al., 2016
	*Homo sapiens*	Histatin-5	Osmotic stress	*Candida sp.*	Puri and Edgerton, 2014
	*Ixodes Ricinus*	DefMT3, DefMT5, DefMT6	Membrane disruption	*Fusarium graminearum, F. culmorum*	Tonk et al., 2015
	castor bean tick				
	*Apis mellifera* royal jelly	Jelleine-I	Increased production of ROS and binding with genome DNA	*Candida sp.*	Jia et al., 2018
	*Avicularia juruensis* spider venom	Juruin	Inhibit voltage-gated ion channels	*Candida* spp., *Aspergillus niger*	Ayroza et al., 2012
	*Bos taurus*, *Homo sapiens*	Lactoferrin B, Lactoferrin H	Plasma membrane disruption	*Candida albicans*, C*andida glabrata*	Fernandes and Carter, 2017, Komatsu et al., 2015
	*Lycosa erythrognatha*	LyeTx I	Aleration of permeabilisation of L-alpha-phosphatidylcholine-liposomes	*Candida krusei*, *Cryptococcus neoformans*	Santos et al., 2010
	spider venom				
	*Pseudopolybia vespiceps* wasp	Mastoparan Polybia-MPII	Membrane pore-formation	*Cryptococcus neoformans*, *Candida albicans*	Silva et al., 2017
	*Sphodromantis viridis* African mantis	Mastoparan-S	Membrane pore-formation	*Fusarium culmorum, Aspergillus niger, A. fumigatus*	Zare-Zardini et al., 2015
	*Vespa tropica* wasp	Mastoparan-VT3, VT-5	Membrane pore-formation	*Candida parapsilosis*	Hong-Ling et al., 2016
	*Megophrys minor* toad	Megin 1 and 2	NA	*Candida albicans*	Yang et al., 2016
	*Phyllomedusa sauvagei -* waxy monkey tree frog	Dermaseptin-S1	Pore-formation	*Candida albicans*	Belmadani et al., 2018
	*Drosophila melanogaster*	Metchnikowin	Cell wall synthesis (interaction with β(1,3)-glucanosyltransferase Gel1)	*Fusarium graminearum*	Moghaddam et al., 2017
	fruit fly				
	*Ornithoctonus hainana* spider venom	Oh-defensin	NA	*Candida albicans*	Zhao et al., 2011
	*Apis mellifera* Bee venom	Melittin	membrane permeabilization, apoptosis induction, inhibition of (1,3)-β-D-glucan synthase, alterations in fungal gene expression	*Aspergillus, Botrytis, Candida, Colletotrichum, Fusarium, Malassezia, Neurospora, Penicillium, Saccharomyces, Trichoderma, Trichophyton, and Trichosporon*	Memariani and Memariani, 2020
	*Eupolyphaga sinensis* cockroach	Es-termicin	Growth inhibition	*Candida parapsilosis,*	Liu et al., 2016
				*Candida albicans*	
	*Synoeca surinama* wasp	Synoeca-MP	Membrane disruption	*Candida* sp.	Freire et al., 2020
	*Podisus maculiventris*	Thanatin	Cell lysis	*Alternaria brassicicola, Fusarium culmorum*	Dash and Bhattacharjya, 2021
	spined soldier bug				
	*Vespa tropica* wasp	VCP-VT1	Membrane disruption	*Candida parapsilosis*	Yang et al., 2013

NA: Not available

### Emerging fungal targets

**Figure 3:**
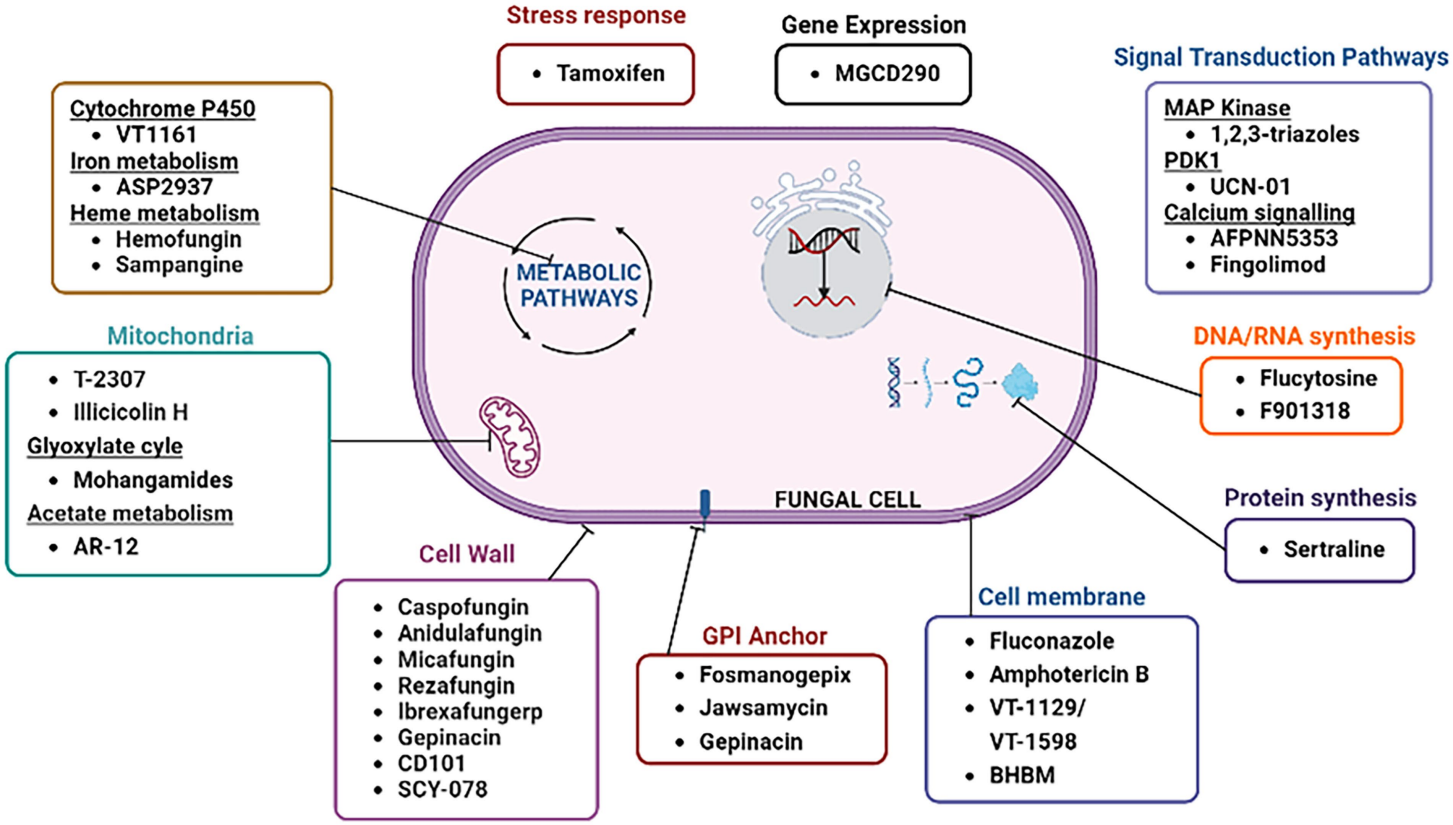
Classical and emerging antifungal targets.

Figure 3 depicts the old as well as emerging fungal targets along with their respective antifungal agents that are either currently available for use or undergoing clinical trials. Some of the fungal targets that have recently gained attention of researchers to develop novel antifungals are described below.

### Glycosylphosphatidylinoitol-anchored proteins

An interesting potential target for developing novel antifungal drugs is the covalent attachment of proteins to glycosylphosphatidylinositol (GPI), which anchors them to the plasma membrane (Yadav and Khan, 2018). The Eisai Company in Japan developed the novel antifungal drug E1210 (fosmanogepix or APX001), which inhibits an initial stage of the GPI-dependent anchoring of mannosylated cell wall proteins linked to the polysaccharide wall component; the cell wall becomes weaker as a result of this interruption, which stops the growth of fungi. The fourth enzyme in the GPI-anchoring pathway, Gwp1p, which is in charge of inositol acylation, is the target of E1210 (the mammalian homolog is not inhibited; Watanabe et al., 2012). *In vitro* tests showed that E1210 was highly effective against the majority of fungi, including strains that were resistant to triazoles and polyenes as well as yeasts (*Candida* species other than *Candida krusei*) and molds (*Aspergillus* species, *Fusarium* species, and black moulds; Miyazaki et al., 2011; Pfaller et al., 2011). In a recent study, fosmanogepix was also found to be effective against Fusariosis in immunocompromised mice (Alkhazraji et al., 2020). A second GPI inhibitor named gepinacin (Phenoxyacetanilide), which is a monocarboxylic amide with antifungal activity against various yeasts and molds was found (Ivanov et al., 2022). An essential acyltransferase needed for the manufacture of fungal GPI anchors, Gwt1, is selectively inhibited by gepinacin (McLellan et al., 2012). The GPI anchor synthetic pathway enzyme Mcd4 is inhibited by M720, a phosphoethanolamine transferase-I inhibitor with *in vivo* activity against *Candida albicans* (Mann et al., 2015). The SPT14 gene (YPL175W), which encodes the fungal UDP-glycosyltransferase catalytic subunit known as Spt14, is crucial for the production of GPI. A natural substance called jawsamycin (FR-900848) contains oligomeric cyclopropyl that targets the catalytic component Spt14 (Fu et al., 2020). Jawsamycin targets the fungal Spt14 but not the human PIG-A, although the human homolog PIG-A and the *S. cerevisiae* Spt14 share 40% similarity (Fu et al., 2020). Jawsamycin demonstrated antifungal action with broad-spectrum and potency against Mucorales fungi, such as *Rhizopus oryzae* and *Absidia corymbifera*, as well as *Fusarium* species, *Scedosporium* species, and *Mucor circinelloides* (Fu et al., 2020).

### Enolase and host plasminogen

Enolase catalyzes the conversion of 2-phosphoglycerate (2-PG) and phosphoenolpyruvate in the penultimate phase of glycolysis (Ji et al., 2016). A genetic knockout of enolase in *C. albicans* has been demonstrated to decrease germination tube and hyphal development, leading to decreased virulence and growth rate, which is consistent with its participation in core metabolic activities (Ji et al., 2016). It’s interesting to note that while this enzyme performs its glycolytic functions in the cytoplasm, many bacterial and fungal species also express it on their cell surfaces. This has been seen in *Aspergillus flavus*, *A. terrus*, *A. nidulans*, *Candida glabrata*, *Saccharomyces* cerevisiae (Funk et al., 2016). The potential of *A. fumigatus* surface-expressed enolase to interact with human immunological regulators, such as factor H, factor-H-like protein 1 (FHL-1), C4b-binding protein (C4BP), and plasminogen, was demonstrated in a thorough investigation (Dasari et al., 2019). When human A549 epithelial cells or an epithelial monolayer were exposed to swollen *A. fumigatus* conidia covered with human plasminogen, cell retraction, as well as cellular metabolic activity, was observed to be lowered significantly (Dasari et al., 2019). The structure-guided design of a peptidomimetic or small molecule inhibitor that precisely targets the enolase plasminogen docking site is a potential route for novel therapies (Dasari et al., 2019). A thorough understanding of the interactions between the two proteins is necessary for this method. Although full-length human Type II plasminogen (4DUR) and fungal enolase from *Saccharomyces cerevisiae* (3ENL) have high-resolution crystal structures, the interface for their interactions has not yet been established (Law et al., 2012).

### Mannitol biosynthesis

A vital mechanism that promotes fungal virulence and survivability is mannitol production. Mannitol is acyclic, six-carbon sterol alcohol that is produced by the major biosynthetic enzymes mannitol-2-dehydrogenase (M2DH) and mannitol-1-phosphate 5-dehydrogenase (M1P5DH). Mannitol is a molecule that is found in almost all the structures of fungi, including mycelia, fruiting bodies, and conidia. It functions as a molecule that stores carbohydrates, an osmolyte, and a source of reducing power (Meena et al., 2015). Mannitol synthesis is boosted during pathogenesis to take advantage of its capacity to squelch reactive oxygen species (ROS) and evade host defenses (Wyatt et al., 2014; Meena et al., 2015). Interest in the underlying mechanism of mannitol’s manufacture and secretion during fungal infection has increased because of the special and varied features of mannitol, particularly concerning the human disease. The fact that humans do not manufacture mannitol and hence lack corresponding enzymes adds to the appeal of targeting mannitol production pathways in fungus. The discovery and development of inhibitory drugs that selectively target these fungal enzymes are made much easier by the lack of a human equivalent.

### Nucleotide biosynthesis

Due to its involvement in vital activities like DNA and RNA synthesis, energy metabolism, and signal transmission, disruption of the purine and pyrimidine biosynthesis pathway using small molecules has been investigated as a potential strategy to produce antimicrobials (Trapero et al., 2018). It is crucial in the drug design process to achieve species-selective targeting of the biosynthesis enzymes since many of these are shared by humans, fungi, and bacteria (Chitty and Fraser, 2017). Thus, the creation of high potency and selectivity inhibitors will benefit greatly from a thorough understanding of the structural and kinetic properties of the fungal and human homologs of these enzymes. A novel substance known as ECC1385 that was discovered in a library of synthetic compounds has been demonstrated to inhibit *C. neoformans* and *C. albicans* GMP synthase activity (Rodriguez-Suarez et al., 2007). It exhibited whole cell action against a wide range of fungi and bacteria *in vitro*, including various species of *Candida*, *A. fumigatus*, *C. neoformans* and *Staphylococcus aureus* (Rodriguez-Suarez et al., 2007). Antiretroviral medicine azidothymidine (AZT; brand name RETROVIR) is being used to treat HIV demonstrated antifungal efficacy *in vivo* and *in vitro* for *A. flavus* (Wang X. et al., 2019). Since AZT has FDA approval, there is potential to employ the AZT scaffold to enhance its antimycotic capabilities or to repurpose it as an antifungal medication. F901318 (olorofim), which was found using an *Aspergillus nidulans* library screen, inhibits the enzyme dihydroorotate dehydrogenase (DHODH), which is responsible for catalyzing the fourth enzymatic stage of pyrimidine biosynthesis (Oliver et al., 2016). Despite being ineffective against Mucorales and *Candida* species, F901318 is quite effective *in vitro* against triazole-resistant *Aspergillus*, *Scedosporium*, *Madurella*, and *Fusarium* species (Law, 2016). Mammalian cells also have DHODH, however, F901318 is unable to inhibit the human version of the enzyme. In addition, olorofim exhibits effectiveness both *in vitro* and *in vivo* against *A. fumigatus* isolates that are resistant to other antifungal medications (Du Pré et al., 2018). The FDA designated olorofim as a “breakthrough drug therapy” in November 2019. It was subsequently given the orphan drug designation for the treatment of invasive aspergillosis, *Lomentospora/Scedosporium* infection (March 2020), and coccidiosis (June 2020), and just recently became an approved medication for the treatment of infectious diseases (June 2020; Wiederhold, 2020).

### Hsp-90

In eukaryotes, Hsp90 is a crucial molecular chaperone that controls the proper transport, maturation, and destruction of client proteins. In addition, Hsp90 controls the growth of mycelium, which is essential for virulence. Drug-resistant fungi are becoming an increasingly serious problem, and Hsp90 may offer a fresh way to address this growing danger (Arastehfar et al., 2020). Hsp90 impacts the survival, pathogenicity, and drug resistance of many diseases. The nucleotide-binding domains (NBD) of Hsp90 are conformationally flexible, according to research by Whitesell L. and colleagues in which they created CMLD013075, which had a more than 25-fold binding selectivity for fungal Hsp90 even though Hsp90 is extremely conserved (Huang et al., 2019; Whitesell et al., 2019). In contrast to previous Hsp90 inhibitors, CMLD013075 demonstrated little toxicity to mammalian cell lines (Whitesell et al., 2019). When used against resistant clinical isolates of *Candida albicans*, CMLD013075 was able to reduce the development of *Candida albicans,* and also improved the antifungal effects of azole antifungals. As a highly selective fungal Hsp90 inhibitor, CMLD013075 is a successful illustration of how IFIs can be treated by preventing fungal Hsp90 from doing its job. Histone deacetylase 2 (Hos2) controls the deacetylation of Hsp90, which is necessary for the interaction of Hsp90 with specific co-chaperones and the stability of several Hsp90 client proteins (van Daele et al., 2019). MGCD290 is a fungus Hos2 inhibitor created by MethylGene, Inc. of Montreal, Canada which not only prevents the deacetylation of histone proteins but also that of non-histone proteins like Hsp90 (Pfaller et al., 2015). Although MGCD290 only exhibits mild anti-*Candida* activity it exhibits good synergistic antifungal activity with azoles against a variety of *Candida* species strains that are resistant to several drugs in an *in vitro* setting (Pfaller et al., 2015). To increase the antifungal effectiveness of antifungal medications against drug-resistant pathogenic fungi, MGCD290 can be used as an adjuvant. It is necessary to confirm the antifungal efficacy of MGCD290 utilizing additional animal models of IFIs and, ultimately, in carefully planned clinical studies.

### Chitin synthase

Chitin is produced by a family of membrane-localized chitin synthase enzymes and is necessary for cell survival (Chs1 to 3, and Chs8 in *C. albicans*). Due to their structural similarity, polyoxins and nikkomycins are powerful inhibitors of the Chs enzyme that compete with the Chs substrate UDP-GlcNAc for Chs binding, although they only have a little impact on whole cells (Shubitz et al., 2014; Ibe and Munro, 2021). *Streptomyces tendae* and *Streptomyces ansochromogenes* both generate the structurally related antifungal drugs nikkomycin X and nikkomycin Z, which have activity against a range of human fungal infections. The respiratory infections caused by *Coccidioides* species can be treated with nikkomycin Z (Shubitz et al., 2014). Recent research has led to the development of two new nikkomycin analogs, nikkomycin Px and Pz, which share the same antifungal properties as nikkomycin X and Z but exhibit significantly greater pH and temperature stability (Feng et al., 2014). Despite having specialized roles, the enzymes involved in the manufacture of chitin might become redundant in certain circumstances. The creation of effective chitin synthase inhibitors has also been hampered by minute variations in protein structure across members of the Chs family.

### Future perspectives and conclusion

The lack of access to specific diagnostic tests and the restricted availability of antifungal drugs are the major challenges faced by clinicians to manage drug-resistant pathogens in present times. Also, the vast diversity of the fungal kingdom ensures an endless supply of new pathogens as well as new variants of known pathogens that are capable of adapting and evolving resistance themselves when exposed to antifungal agents. The key issue is that mostly *in-vitro* experiments have been used to investigate the activity of newly discovered antifungals. As the novel antifungal research moves forward, more studies using *in-vivo* infection models and subsequent clinical studies are required. Nevertheless, several naturally available agents all around us show promise in fending against the growing threat posed by fungi. There has been significant progress in identifying possible targets for new agents that take advantage of intrinsic dissimilarities between humans and fungi. Even while many of these targets are still hypothetical, some compounds have just recently started their early stages of clinical research. By increasing funding for research, exploring unexplored as well as underexplored sources of natural antimicrobials, taking into account novel alternative targets, repurposing already-existing non-antifungal medications, fortifying ties between academia and industry, and offering financial incentives for the development of new therapeutic options to treat these uncommon but deadly infections, we must address this unmet need.

## Author contributions

MC and BN: data curation, writing–original draft preparation, and reviewing and editing. VijK: conceptualization, writing– original draft preparation, reviewing and editing, and supervision. AV: original draft preparation and reviewing, and editing. PS: original draft preparation, reviewing, and editing. VivK and SG: original draft preparation, reviewing, and editing. All authors contributed to the article and approved the submitted version.

## Conflict of interest

The authors declare that the research was conducted in the absence of any commercial or financial relationships that could be construed as a potential conflict of interest.

## Publisher’s note

All claims expressed in this article are solely those of the authors and do not necessarily represent those of their affiliated organizations, or those of the publisher, the editors and the reviewers. Any product that may be evaluated in this article, or claim that may be made by its manufacturer, is not guaranteed or endorsed by the publisher.
